# Exploring Vaccination Challenges among Syrian Refugees in Jordan: Insights from Camps and Communities, and Perceived Parental Barriers to Childhood Vaccination Uptake

**DOI:** 10.3390/vaccines12020133

**Published:** 2024-01-27

**Authors:** Bayan Abdulhaq, Muhammad Hammouri, Dania Abu Hawas, Latefa Ali Dardas

**Affiliations:** 1 School for International Training, Brattleboro, VT 05302, USA; 2 The University of Jordan, Amman 11942, Jordan

**Keywords:** vaccination challenges, refugees, parental barriers, vaccination uptake

## Abstract

Purpose: To identify and understand the multifaceted barriers faced by Syrian refugees when seeking vaccination services for their children. Methods: A survey questionnaire was administered through structured interviews to a sample of Syrian refugees residing inside the Al-Zaatari camp and in various urban areas across Jordanian communities. This process utilized a multi-stage sampling approach, beginning with a random selection from clusters or strata, and then employing convenience sampling within each to select participants. The survey covered demographics, barriers to vaccination, and vaccine hesitancy. Results: A total of 332 participants completed the survey with a mean age of 32.7 ± 10 years ranging from 18 to 67. More than half of the sample (59%) had an education of 11th grade or less. Sociodemographic disparities regarding barrier perception were evident among participants. Middle-aged adults (older than 32), males, and those with a monthly income less than USD 200 had scored significantly higher on barrier perceptions across all categories (*p* < 0.05). In-camp residents were less likely to face vaccination barriers compared to those living outside the camps (*p* < 0.001). Psychological antecedents of vaccine assessments showed that younger individuals had significantly higher scores in complacency, calculation, and constraints (*p* < 0.05). Participants with lower income had lower constraints and calculation scores (*p* < 0.05). In-camp residents had significantly higher scores in complacency, constraints, and calculation constructs compared outside camps counterparts (*p* < 0.05). Participants with no formal education had higher scores in complacency and constraints, and those with less than a 12th-grade education and higher education degrees scored significantly higher on the collective responsibility construct (*p* < 0.05). Conclusions: Efforts to promote vaccination among refugees should consider the specific challenges faced by this population, including financial barriers, healthcare access inequalities, and the impact of living arrangements. Public health strategies should address not only individual and psychological factors but also the physical and logistical challenges in obtaining vaccines.

## 1. Introduction

The ongoing evolution of migration dynamics demands a heightened focus on addressing pressing public health concerns, particularly in the context of infectious disease transmission. Recent estimates from the United Nations High Commissioner for Refugees (UNHCR) indicate that around 89.3 million people are forcibly displaced worldwide, of which 27.1 million are refugees [1]. This unprecedented scale of displacement has brought to the forefront a critical and often overlooked challenge—the vaccination status of newly arrived migrants. Research conducted by Napoli and colleagues [2] has shed light on the prevailing gap in structured vaccination status screening among this vulnerable demographic. This dearth of systematic screening, coupled with a lack of comprehensive guidance, underscores the urgent need for further investigation into vaccination status and the multifaceted issues that may hinder acceptance and uptake among migrant populations. Such efforts can substantially contribute to early detection, treatment, and prevention of vaccine-preventable diseases (VPDs) among migrants, with far-reaching implications for both the health of migrants themselves and the broader public health landscape.

In the Middle East in particular, geopolitical conflict and persecution have led to extensive forced displacement of millions of civilians from many countries to other neighboring nations [3]. Most notably, the recent Syrian crisis is considered by the United Nations to be the worst refugee catastrophe since the Second World War with more than 5 million displaced individuals across Jordan, Lebanon, and Turkey [4]. These populations from areas of conflict are confronted with considerable and complicated healthcare challenges that often create fertile grounds for the spread of infectious diseases and malnutrition among refugees [5,6,7]. In fact, studies have consistently shown that refugees have a higher burden of said diseases compared to their host populations [8]. Studies from Western countries show that bypassing these health disparities and health inequality is hindered by many barriers, namely, refugees’ nativity, language divide, background and residential duration, generation status, and cultural and persona beliefs in addition to age, gender, maternal education, and socioeconomic status [8,9]. These barriers further extend to include provider- and structure-related such as that of poor physician–patient communication, and the dearth of adequate public health communication strategies to educate about vaccinations [9,10,11].

Within the context of the Middle East and Jordan, the literature is lacking in describing barriers to achieving optimal vaccination programs for the refugee population. Some barriers may be specific to the region such as that of religiosity or religious beliefs which are often not studied in the West. Apropos to that, measles coverage was as low as 54% in more religious areas in Aceh Province, an autonomous region that has instituted Sharia law in Indonesia [12]. Furthermore, healthcare access inequalities in those regards were more evident in Muslim women compared to their Hindu male counterparts [13]. It is important to note that studies have resurfaced to emphasize this challenge, especially in Jordan, as the continued influx of populations from conflict-affected areas and the low immunization coverage in many parts of the country could predispose it to outbreaks in the future [14]. Moreover, differences in living arrangements between refugee populations in Jordan present with unique challenges that are often not addressed in the literature. As the majority of Syrian refugees live in urban settings among host communities in Jordan with only 20% living within camp borders, there are stark differences in encountered obstacles relating to healthcare and education between both settings [15,16]. For example, although in-camp residents have relatively easier access to basic needs, barriers relating to limited employment opportunities and restricted movement due to lack of transportation are some of the downsides they experience in comparison to out-camp living, which, in of its own, has its distinct set of hardships [17].

In the midst of the increasing significance of addressing health equity among refugee populations, it is noteworthy that a comprehensive examination of the impediments obstructing access to vaccination programs among Syrian refugees in Jordan has remained absent. This study seeks to bridge this gap by embarking on identifying and understanding the multifaceted barriers that Syrian refugees in Jordan confront when it comes to seeking vaccination services for their children. The insights gleaned from this research hold the potential to inform the development of targeted and effective VPDs policies and interventions. By tailoring these initiatives to the specific health challenges experienced by Syrian refugees in Jordan, especially those related to parental concerns and beliefs about childhood vaccinations, we aspire to foster a more equitable and accessible healthcare environment for this marginalized group. The recognition of parental perspectives in this context is pivotal, as it not only impacts individual child health but also plays a crucial role in community-level immunization rates. By addressing perceived parental barriers, we aim to empower parents with the information and support needed to make informed decisions regarding childhood vaccinations, ultimately contributing to the broader mission of promoting health equity among those who have been disadvantaged by the complex circumstances of displacement and migration.

## 2. Methods

### 2.1. Design

The survey design for this study aimed to comprehensively investigate the barriers to vaccination access among Syrian refugees in Jordan, both within Al Zaatari camp and in the Northern and Southern regions of the country. According to Aguilera et al. [18], designing a survey to ensure the collection of a representative sample of refugees is often challenging due to the lack of updated and comprehensive sample frame. In response to this challenge, we adopted the approach of Aguilera et al., which involved two key innovations. The first innovation centered around using mutually exhaustive and exclusive sampling units of roughly equal population size when available. In our case, this could be applied with Al Zaatari camp, where a multi-stage sampling approach was employed. Inside the camp, the population was divided into clusters representing its 12 districts. From each randomly selected cluster, a convenience sample of individuals participated in the survey.

However, for refugees outside camps, we utilized the second innovation involved leveraging available information from various sources on the prevalence of Syrian refugees in Jordan, including mainly those provided by the UNHCR in its quarterly analysis 2023 [19]. While this information was often available at a higher geographic level than the smaller sampling units we intended to use, it proved invaluable in estimating known probabilities of selection. A stratified random sampling method was adopted, stratifying by geographical region. Communities were randomly selected within each stratum, and a convenience sample participated in the interviews. Combining these innovative approaches aimed to overcome the challenge of establishing a representative sample frame for our survey of Syrian refugees in Al Zaatari camps and communities across North, Central, and South Jordan. 

Structured interviews conducted via iPads were the primary method of data collection, ensuring standardized data collection procedures. A team of 12 trained research assistants conducted the interviews, and data quality checks were performed to identify and rectify any errors. Participants were in healthcare centers as well as through home visits. Participants older than 18 years of age and who were able to consent and comprehend questions asked within the interviews were included. The study received approval from the University of Jordan institutional review board.

With the lack of a reliable and updated sampling frame, we calculated the sample size using a formula-based approach, such that the required sample size = [(Z2 × p ∗ (1 − p))/E^2^]. Where the Z-score was 1.96, p is the estimated proportion set at 50%, which is recommended with the population size is unknown, and E is the margin of error estimated at 5%, resulting in a sample size requirement of approximately 385 respondents to meet the specified confidence and margin of error criteria. In order to ensure a balanced representation of both populations residing inside and outside the camps, our study aimed to secure a total sample size of 385 respondents, equally distributed between these two groups, accounting for approximately 50% within the camps and 50% outside the camps.

### 2.2. Measures 

A comprehensive survey questionnaire was developed, covering three key aspects:

1. Demographics, including their gender, age, marital status, educational level, income, the number of children, residence status, occupation, current health problems, child health problems, and the likelihood of seeking help in case of health concerns.

2. Barriers to Vaccination: The second section consisted of 61 items, each rated on a Likert scale ranging from 1 (strongly disagree) to 7 (strongly agree). These items were designed to assess the perceived barriers to vaccination among the participants. These barriers were categorized into six distinct categories, adapted from Kaufman’s et al. global review of systematic reviews on parent-level barriers to uptake of childhood vaccination [20]. These included: (1) Access barriers, assessing issues related to physical access and availability of vaccination services. (2) Clinic or health system barriers, examining barriers associated with the healthcare system itself. (3) Concerns and beliefs, exploring participant concerns, beliefs, and attitudes towards vaccination. (4) Health perceptions and experiences barriers, evaluating how personal health perceptions and experiences impact vaccination decisions. (5) Knowledge and information barriers, assessing barriers related to information and knowledge about vaccines. (6) Social or family influence barriers, examining the influence of social and family factors on vaccination decisions. The adapted questionnaire was piloted and subsequently underwent a face validity assessment conducted by experts in the field.

3. Vaccine hesitancy: This section included a 15-item tool derived from the “5C model” of psychological factors influencing vaccination [21]. Each of the five factors—confidence, complacency, constraints, calculation, and collective responsibility—was evaluated through three rating items on a 7-point scale (1 = strongly disagree; 7 = strongly agree). The average scores of items within each category were calculated, with a higher mean score indicating a higher level of agreement with that particular factor. The tool was translated into Arabic and validated with Arab refugees and host communities [22,23,24].

### 2.3. Statistical Analysis

Data were analyzed using SPSS^®^ version 23. Categorical variables were reported as frequencies and percentages and continuous variables were reported as mean ± standard deviation. For items rated on a 7-point Likert scale, responses were grouped into three categories that are agree, disagree, or are neutral for simplicity of reporting. Differences in perceptions of both barriers and the 5C domains were examined using either the *t*-test or ANOVA depending on the categorical variable nature. Gender, income level, and residence in camps were examined using the *t*-test, while educational level was the only variable examined using ANOVA(version 23). Due to little discrimination in some demographics, variables such as income level (6 groups) and educational level (5 groups) were re-categorized in 2 and 4 groups, respectively. This was conducted in order to increase the statistical power of conducted tests. All statistical tests are conducted with a 95% confidence interval and a 5% error margin. A *p*-value of less than 0.05 is considered statistically significant.

## 3. Results

### 3.1. Participants 

A total of 332 participants completed the study questionnaire. The recruited sample had a mean age of 32.7 ± 10 years ranging from 18 to 67. Of them, 50.3% resided inside a camp, while 49.7% lived outside camp settings. The majority (82.5%) were female and reported being married (93.7%), with 59% having an educational background of 11th grade or lower. Additionally, 18.3% were employed. About 22% acknowledged having health issues (22.5%). Moreover, child health problems were reported by 13.2% of our sample. In contrast, only a small fraction had sought help for health concerns previously (5.7%). Table 1 details participants’ characteristics.

### 3.2. Barriers to Vaccinations

**Figure 1:**
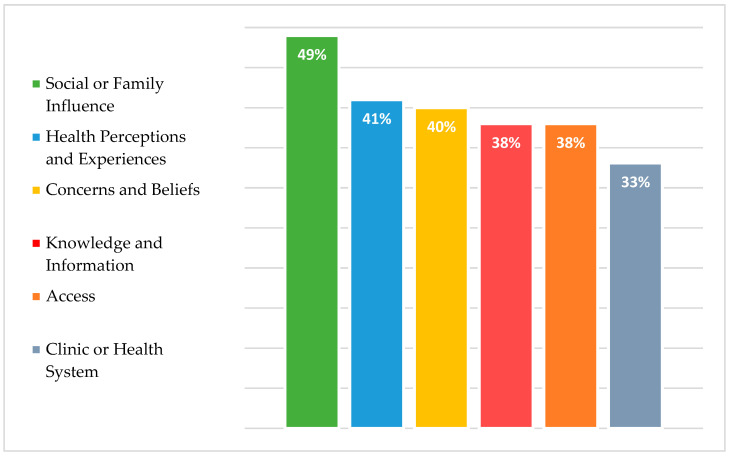
Perceived parental barriers to childhood vaccination uptake among Syrian refugees.

Across the six broad categories, the most commonly reported perceived barriers by our cohort were primarily linked to social and family influence (48.9%), closely followed by perceptions about one’s health and vaccines (40.5%), and concerns and beliefs regarding immunization (40.4%). Additionally, lack of information and proper knowledge (38.2%), difficulties in accessing healthcare (38%), as well as various barriers related to the healthcare system and facilities (33.1%) were also reported (refer to Figure 1). 

The most commonly cited barriers in each category were as follows; in terms of social or family influence, the main barrier was related to gender norms regarding responsibility of child vaccination follow-up schedules (50.9%). As for accessibility barriers, having trouble securing care for other children (27.7%), facing long waiting times at clinics (24.7%), and the lack of comprehensibility of health systems (24.7%) were the most agreed upon barriers in our population. As for barriers related to healthcare system, clinic delays, disorganization of health facilities (16.2%), as well as fear of disease exposure due to subpar quality of healthcare facilities and used equipment (16%) were the most commonly encountered, as reported by our participants. In terms of concerns and beliefs, more than one third of our sample agreed that parents should have full autonomy in deciding or rejecting vaccines (37.9%). Feelings of regret and hesitancy about future vaccines when side effects of current vaccines occur was also encountered by one third of our sample (33.7%). Regarding health perceptions and experiences, half of participants stated that their children had contraindications that prevented them from receiving vaccinations (49.9%). Moreover, one third of our sample believed that there is no need to take the vaccine due to the mild severity of illness or invulnerability of children (30.1%). Finally, for knowledge and information related barriers, lack of awareness about primary healthcare facilities’ location or was the most commonly reported barrier (19.9%) with insufficient knowledge about vaccine doses and schedules coming at a close second (18.4%). Figure 1 delineates barriers reported by participants across different categories. Table 2 showcases responses to vaccination barriers in their respective categories. 

### 3.3. Sociodemographic Differences in Barrier Perception

Our cohort demonstrated that male participants, individuals older than 32 years (sample mean age), those with a monthly income of less than JD 150 (USD 211) had scored significantly higher across all barriers categories compared to their counterparts (all *p* < 0.05). Furthermore, In-camp residents were less likely to report facing vaccination barriers compared to refugees living outside camp borders (*p* < 0.001). With regard to marital status, with the exception of access barriers, married individuals were less likely to report obstacles related to immunization across all barriers categories (*p* < 0.05). In terms of educational level, participants with secondary education reported the highest barrier score with regard to accessibility, vaccination concerns and beliefs, and social or family influence (*p* < 0.001). Participants with higher education reported higher barrier scores in terms of vaccine knowledge, perceptions and health experience, and barriers related to the healthcare system (*p* < 0.05). Detailed barrier scores stratified according to relevant factors could be found in Table 3 and Table 4.

### 3.4. Psychological Antecedents of Vaccination Adoption

As for the constructs of the 5C model, female participants scored higher on complacency, constraints, and calculation constructs (*p* < 0.05). No significant differences were noted in collective responsibility and confidence between both genders. Younger individuals had significantly higher complacency, calculation, and constraints construct scores (*p* < 0.001). Participants with lower income had lower constraints and calculation mean scores compared to those relatively higher income (*p* < 0.05). In-camp residents had significantly higher mean score in complacency, constraints, and calculation constructs compared to refugees living outside camps (*p* < 0.001). Participants with no formal education had higher mean scores on the complacency and constraints constructs (*p* ≤ 0.001). Respondents with less than 12th-grade education and those who held higher education degrees scored significantly higher on the collective responsibility construct (*p* < 0.05). Table 3 and Table 4 show detailed differences between 5C construct scores across various sociodemographic variables.

## 4. Discussion

Our study sought to establish a baseline for the unique challenges and barriers faced by Syrian refugees in Jordan on multiple facets and their correlation to sociodemographic variables of participants. Furthermore, this is the first comprehensive report of the obstacles encountered by the displaced populations in Jordan with regard to access to immunizations, to the best of our knowledge. 

We identified several barriers relating to accessibility to healthcare, quality of available health facilities, participants’ own convictions and knowledge regarding vaccines and health, and barriers relating to peer influence. These categories correspond well with the body of reported literature on the subject [25,26,27]. The significant growth in studies in this field over time reflects the increasing importance and relevance of the implications of large-scale migration and recent refugee crises and their potential role in the re-emergence of VPDs. Despite the overall increase in the number of studies, most were conducted in high income countries [28]. Reducing VPDs among the refugee population requires well-informed strategies addressed to their needs to improve vaccination rates. This becomes crucial considering the low rates of immunizations in Syrian refugees in Jordan [24].

Of the barriers mentioned in our study, those penitent to personal factors relating to vaccines and health perceptions (i.e., what people feel and think about their health and vaccines) were among the most commonly cited by our participants. These factors often times entail safety concerns and vaccine side effects and are often reported as key drivers to vaccine hesitancy in the refugee population. A study of 1037 Syrian refugees regarding COVID-19 vaccine refusal found significant associations with negative perceptions regarding their safety profile [29]. Perceived risks have also been shown to be important factors when making immunization decisions. Perceived dangers of the vaccine are often weighed against whether they need it or the possible hazards relating to VPDs [30,31]. Trust in the vaccination process itself and the healthcare system is furthermore a heavy influencing factor regarding immunization. Such beliefs are partly rooted in negative experiences of healthcare systems in their host or home countries [32].

Individual awareness and access to information are often key determinants in vaccine hesitancy. A better understanding of vaccines and a wider access to high quality information are closely related to health and digital literacy which, in return, governed by many sociodemographic variables. Predictors of higher barrier scores relevant to individual knowledge and concerns about immunization were significantly associated with older age and low income. These may be heavily related to lack of proper information access related to technologic and digital literacy [33,34]. Factors influencing vaccine uptake are closely related to same elements affecting other health inequalities and social determinants of health such as that of education level of the parents and income [35,36,37].

One-fourth of our participants declared having financial barriers relevant to decreased vaccine uptake. Economic barriers and vaccine affordability have long been postulated as key factors in driving decision making in refugees regarding immunization. These are related to direct costs related to the vaccine itself and indirect costs of transport and payment loss from time off work. These results parallel those from other reports as studies indicate that refugees are less likely to immunize if costs are increased [38]. A qualitative report in the United States described refugees and migrants having more favorable attitudes towards HPV vaccination as long as it was within financial reach [39]. In Jordan, free universal healthcare coverage for Syrian refugees was implemented initially until 2014. Between 2012 and 2013, however, the number of physicians per 10,000 decreased significantly from 27.1 to 20.2 primarily due to the arrival of more than half a million Syrian refugees at the time. In light of the unsustainable healthcare costs and the increased burden, the Jordanian Ministry of Health ceased offering full healthcare coverage in September of 2014 [15]. Nowadays, the UNHCR covers the entirety of primary and secondary healthcare expenses for refugees referred from within camps. It is worth mentioning, nonetheless, that 80% of refugees reside outside camps in urban areas of Jordan. Those living in such conditions are obligated to pay foreigner’s fees at governmental clinics. The average out-of-pocket healthcare expenditure for Syrian refugees in Jordan was around USD 80 in 2014, a significant amount considering an average household income of around USD 300 which correspond to JD 56.75 and 212.8, respectively [40,41,42].

The impact of living arrangements (i.e., in-camp versus out-camp) among refugees has long been an argument of both sides. On one hand, in-camp refugees often have stronger healthcare systems providing recreational programs for kids, mental health facilities, home visits conducted by field officers to ensure proper care for many chronic illnesses, and penitent to our discussion, many vaccination awareness campaigns. Poor living conditions and restrictions on many aspects, however, lead more than 60% of refugees to live outside of camps where although higher quality of life for them is achieved, challenges of high costs of living and limited healthcare services are met [15,16,17,43,44]. Our results reflect well with the aforementioned as many living outside camps have declared facing barriers much more than their in-camp counterparts.

With regard to the ‘5C’s’ model, younger individuals, higher income, and those living in-camps were all positive predicting variables to having higher scores across its domains, which corresponds with favorable behaviors towards vaccines. The 5C model serves as a novel tool to measure psychological antecedents to vaccinations among individuals and has been adopted in many studies [21,45]. However, researchers have emphasized the importance of identifying more precise terminology when describing vaccine hesitancy. Those distinctions between individual and psychological factors on one hand, and those related to physical and logistical challenges in obtaining vaccines should be well addressed as the latter may have drastic influence on the other especially when considering the refugee population [46]. Refugees encounter distinct personal, societal, and physical hurdles when it comes to accessing healthcare and vaccination services. These obstacles may have a significant effect on lowering their motivation and therefore decrease their willingness to participate in immunizations. This may particularly be the case for refugees who are not familiar with the host country’s healthcare system and those residing outside of official camps where healthcare may be relatively more accessible, hence excluding them from mainstream vaccination systems [24,47,48].

Reducing VPD rates among refugees and increasing their vaccination rates requires well-put strategies tackling the different barriers encountered by this population. Multiple studies have shown the positive effects of educational programs implemented in school and community settings in resigning awareness regarding vaccinations [49]. Provider-based interventions, such as that of mass screening, treatment, and vaccination are important measures to undertake to identify high-risk groups [50]. These strategies were discussed to be more effective in camp-like settings due to high population density and limited infrastructure [51]. Utilizing simple technological tools, such as smartphone applications, to better facilitate vaccination appointments and follow-up visits has also been proven its effectiveness [52,53]. Publicly funding vaccination campaigns as well as arranging organized door-to-door immunization programs with local actors and non-governmental organizations have yielded exceptional results with more than 20,000 vaccine doses successfully delivered, studies report [38,39,54]. It is important to note that proper guidelines and implementation of coherent policies are of utmost importance to increase vaccine uptake and reduce potential spread of VPDs. The fundamental principle behind system-based interventions is that by implementing effective control measures, it becomes feasible to decrease or even eradicate disease occurrences resulting from potentially infectious high-risk populations, like refugees, and safeguard the host population against epidemics [55,56].

### Limitations 

Certain limitations related to sample selection should be acknowledged. In the case of Al Zaatari camp, despite random sampling, there were clusters or groups within the camp that were underrepresented due to logistical constraints. The absence of a comprehensive and accurate sampling frame for refugees outside camps potentially compromised the precision of our random sampling. Another important consideration is the reliance on self-reported data, which is inherent to survey-based research. Future studies may explore complementary methods or data sources to further validate and enhance our understanding of vaccination challenges in this population.

## 5. Conclusions

This study sheds light on the perceived parental barriers to childhood vaccination uptake among Syrian refugees in Jordan. It highlights the complex interplay of factors such as accessibility, healthcare perceptions, and socioeconomic conditions that influence vaccination decisions in this population. Understanding these barriers is critical for the development of targeted interventions and policies aimed at improving vaccination rates and reducing vaccine-preventable diseases among Syrian refugees. Efforts to promote vaccination among refugees should consider the specific challenges faced by this population, including financial barriers, healthcare access inequalities, and the impact of living arrangements. Public health strategies should address not only individual and psychological factors but also the physical and logistical challenges in obtaining vaccines. Technology-based solutions, community-based educational programs, and provider-based interventions can play pivotal roles in increasing vaccine uptake. Ultimately, by addressing the perceived parental barriers to childhood vaccination in the Syrian refugee population, we can contribute to achieving greater health equity among those who have been disproportionately affected by the complexities of displacement and migration. This study serves as a foundation for future research and policy development in this important area of public health.

## Figures and Tables

**Table 1:** Participant characteristics and sociodemographic information.

Characteristic	*n* (%)
**Gender**	
Male	58 (17.5%)
Female	274 (82.5%)
**Mean age**	32.7 ± 10
**Marital status**	
Married	311 (93.7%)
Divorced	8 (2.4%)
Widowed	13 (3.9%)
**Educational level**	
No schooling	31 (9.3%)
11th grade or less	196 (59%)
12th grade	53 (16%)
Diploma	16 (4.8%)
Bachelor’s degree or higher	36 (10.8%)
Income (in JD *)	
Less than 150	240 (72.3%)
150–300	62 (18.7%)
301–500	25 (7.5%)
501–1000	2 (0.6%)
1001–1500	3 (0.9%)
1501–2000	0 (0%)
**Number of children**	
0	8 (2.4%)
1–3	165 (49.5%)
4–6	132 (39.6%)
>6	27 (8.1%)
**Residence**	
Inside a camp	167 (50.3%)
Outside a camp	165 (49.7%)
**Occupation**	
Unemployed	271 (81.3%)
Employed	61 (18.3%)
**Health problems**	
Have a health problem	75 (22.5%)
Does not have a health problem	257 (77.1%)
**Child health problems**	
Has a health problem	44 (13.2%)
Does not have a health problem	288 (86.4%)
**Seeking help before**	
Yes	19 (5.7%)
No	313 (93.9%)

* JD 1 = USD 1.41

**Table 2:** Responses to vaccination barriers in their respective categories.

Items Classified by Barrier Category	Disagree n (%)		Neutral n (%)		Agree n (%)	
**Access Barriers**						
(1) I don’t have enough time to get vaccinated.	261	(78.6%)	26	(7.8%)	45	(13.6%)
(2) I have trouble affording financial vaccinations.	243	(73.2%)	26	(7.8%)	63	(19.0%)
(3) I am facing a problem with transportation, and this prevents me from taking vaccinations.	239	(72.0%)	22	(6.6%)	71	(21.4%)
(4) I have a problem because there is no comprehensive health system.	222	(66.9%)	28	(8.4%)	82	(24.7%)
(5) I have a problem with waiting a long time when I receive the vaccine.	220	(66.3%)	30	(9.0%)	82	(24.7%)
(6) I have trouble securing care for my other children when we will get vaccinated	208	(62.7%)	32	(9.6%)	92	(27.7%)
(7) I have a problem with impermanent residence and frequent commuting	244	(73.5%)	25	(7.5%)	63	(19.0%)
(8) I receive inadequate healthcare support due to social exclusion	266	(80.1%)	31	(9.3%)	35	(10.5%)
(9) I have a problem with vaccines availability.	278	(83.7%)	25	(7.5%)	29	(8.7%)
(10) I have appointment time difficulties related to get vaccines.	280	(84.3%)	20	(6.0%)	32	(9.6%)
(11) I don’t have health insurance, and this is preventing me from getting vaccinations.	260	(78.3%)	23	(6.9%)	49	(14.8%)
(12) I have trouble relating to the cost of transportation to a healthcare facility	243	(73.2%)	23	(6.9%)	66	(19.9%)
(13) I have trouble accessing vaccinations for other reasons that have not been mentioned.	269	(81.0%)	35	(10.5%)	28	(8.4%)
**Clinic or Health System Barriers**						
(14) Health providers poor communication constitute an obstacle to obtaining vaccinations	166	(94.3%)	1	(0.6%)	9	(5.1%)
(15) There is no vaccination reminder system.	277	(83.4%)	35	(10.5%)	20	(6.0%)
(16) There is a delay in the clinic and disorganization in its health facilities.	257	(77.4%)	21	(6.3%)	54	(16.3%)
(17) I was not informed enough about vaccination recommendation.	253	(76.2%)	34	(10.2%)	45	(13.6%)
(18) The poor buildings and equipment makes me afraid of exposure to pathogens	252	(75.9%)	27	(8.1%)	53	(16.0%)
(19) Health centers are not adequately funded and monitored.	239	(72.0%)	47	(14.2%)	46	(13.9%)
(20) Lack of culturally appropriate healthcare or linguistic barriers is an obstacle to getting vaccines.	235	(70.8%)	44	(13.3%)	53	(16.0%)
(21) Healthcare providers have negative attitudes and they do not have enough health knowledge.	285	(85.8%)	26	(7.8%)	21	(6.3%)
(22) I can’t get another appointment to get vaccinated when I miss my appointment.	282	(84.9%)	29	(8.7%)	21	(6.3%)
(23) Poor relationship with providers reduces the possibility of going to get vaccinations.	265	(79.8%)	37	(11.1%)	30	(9.0%)
(24) Providers hesitate or are lazy to administer vaccinations.	281	(84.6%)	27	(8.1%)	24	(7.2%)
**Concerns and Beliefs**						
(25) I am concerned about the safety of the vaccines and I’m afraid of their possible damage.	268	(80.7%)	31	(9.3%)	33	(9.9%)
(26) I mistrust vaccinations, their providers, or the health policies in force.	254	(76.5%)	33	(9.9%)	45	(13.6%)
(27) I believe having illnesses strengthen a child’s immune system better than vaccinations.	247	(74.4%)	36	(10.8%)	49	(14.8%)
(28) I believe that vaccinations compromise the immune system.	266	(80.1%)	32	(9.6%)	34	(10.2%)
(29) I believe that there is immune system response variation among children.	177	(53.3%)	38	(11.4%)	117	(35.2%)
(30) I think vaccines are provided when children are too young.	211	(63.6%)	32	(9.6%)	89	(26.8%)
(31) I believe that parents have the full right to accept or reject the vaccination.	167	(50.3%)	39	(11.7%)	126	(38.0%)
(32) I believe that combination vaccines (triple, quintuple) are harmful effects on the body.	220	(66.3%)	34	(10.2%)	78	(23.5%)
(33) I believe that multiple vaccinations and doses are unsafe for the child.	240	(72.3%)	62	(18.7%)	30	(9.0%)
(34) As a mother, I do not have the capacity to take my child to receive vaccines.	224	(67.5%)	48	(14.5%)	60	(18.1%)
(35) I believe that vaccinations have a role in the child’s autism.	261	(78.6%)	29	(8.7%)	42	(12.7%)
(36) I believe that epidemics and vaccinations are an external conspiracy.	279	(84.0%)	40	(12.0%)	13	(3.9%)
(37) I am worried about injection site pain.	211	(63.6%)	47	(14.2%)	74	(22.3%)
(38) I feel regret and guilt if vaccine side-effects occurred.	200	(60.2%)	30	(9.0%)	102	(30.7%)
(39) I believe that there is no flexibility to adapt a vaccine schedule to a child.	234	(70.5%)	29	(8.7%)	69	(20.8%)
(40) I am concerned about vaccinations in general.	243	(73.2%)	45	(13.6%)	44	(13.3%)
**Health Perceptions and Experiences**						
(41) The child has a contraindication on appointment day prevented me from vaccination.	131	(39.5%)	35	(10.5%)	166	(50.0%)
(42) I believe that there is no need to take the vaccine because the disease is not severe.	203	(61.1%)	29	(8.7%)	100	(30.1%)
(43) I am concerned about potential allergies after taking the vaccine.	229	(69.0%)	35	(10.5%)	68	(20.5%)
(44) I think that vaccines are not effective	222	(66.9%)	33	(9.9%)	77	(23.2%)
(45) I prefer alternative healthcare (such as herbal remedies) instead of vaccinations.	270	(81.3%)	42	(12.7%)	20	(6.0%)
(46) I have personal objections to vaccinations.	281	(84.6%)	31	(9.3%)	20	(6.0%)
(47) My child’s fear of needles is an obstacle to getting immunized.	254	(76.5%)	48	(14.5%)	30	(9.0%)
(48) Poor past experiences with health services constitute an obstacle to vaccination.	251	(75.6%)	28	(8.4%)	53	(16.0%)
**Knowledge and Information**						
(49) I believe that there is insufficient knowledge of the importance of receiving all vaccines dose.	260	(78.3%)	38	(11.4%)	34	(10.2%)
(50) I believe that there is insufficient quantity and quality of information related to vaccines.	238	(71.7%)	36	(10.8%)	58	(17.5%)
(51) Insufficient knowledge about the vaccination dose schedule made me not adherent to them.	236	(71.1%)	35	(10.5%)	61	(18.4%)
(52) What I read of vaccine-related information in the media made me not adhere to it.	241	(72.6%)	48	(14.5%)	43	(13.0%)
(53) The available information about the content of the vaccines is not adequate.	249	(75.0%)	35	(10.5%)	48	(14.5%)
(54) Unawareness of vaccination services, clinic location, or the timing, made me not adhere to it.	241	(72.6%)	25	(7.5%)	66	(19.9%)
(55) Uncertainty about the importance of vaccinations made me not adhere to it.	263	(79.2%)	35	(10.5%)	34	(10.2%)
(56) Forgetting the appointments (schedule) for vaccinations made me not adhere to them.	246	(74.1%)	28	(8.4%)	58	(17.5%)
(57) The available information about vaccinations is not appropriate for all education levels.	248	(74.7%)	32	(9.6%)	52	(15.7%)
**Social or Family Influence**						
(58) Religious beliefs regarding vaccinations made me not adhere to them.	246	(74.1%)	40	(12.0%)	46	(13.9%)
(59) I believe that vaccination is a societal duty for community protection.	135	(40.7%)	32	(9.6%)	165	(49.7%)
(60) Following up on vaccination schedules is the mother’s responsibility only.	141	(42.5%)	22	(6.6%)	169	(50.9%)
(61) Diseases can be treated through alternative methods such as going to the clergy.	251	(75.6%)	23	(6.9%)	58	(17.5%)

**Table 3:** Mean barrier and 5C construct score differences across gender, age, and income.

Measures Domains		Gender			Age			Income (in JD +)		
		Male	Female	*p*-Value	<32	>32	*p*-Value	<150	>150	*p*-Value
Barrier	Access	45.3 + 19.1	32.4 + 13.7	<0.001 *	28.5 + 11.7	39.7 + 16.3	<0.001 *	37.1 + 16.8	28.1 + 8.7	<0.001 *
	System	32.3 + 13.7	24.0 + 9.6	<0.001 *	21.7 + 8.3	28.7 + 11.6	<0.001 *	26.4 + 11.7	23.1 + 7.9	0.014 *
	Concerns and Beliefs	56.9 + 19.8	42.7 + 16.7	<0.001 *	39.7 + 15.2	49.4 + 18.8	<0.001 *	46.9 + 19.6	40.7 + 12.5	0.005 *
	Perceptions	33.1 + 11.6	23.9 + 9.6	<0.001 *	22.2 + 9.3	28.4 + 10.9	<0.001 *	26.6 + 11.3	22.7 + 7.7	0.003 *
	Knowledge	28.1 + 12.4	20.0 + 9.6	<0.001 *	18.3 + 8.8	23.9 + 11.4	<0.001 *	22.5 + 11.3	18.6 + 7.7	0.003 *
	Social and Family	15.3 + 4.2	13.3 + 3.8	0.001 *	12.5 + 3.6	14.7 + 3.9	<0.001 *	14.0 + 4.1	12.8 + 3.3	0.013 *
Construct	Confidence	6.7 + 0.2	6.7 + 0.4	0.921	6.7 + 0.4	6.6 + 0.3	0.293	6.7 + 0.3	6.7 + 0.4	0.518
	Complacency	1.0 + 0.1	1.6 + 0.7	<0.001 *	1.8 + 0.7	1.3 + 0.6	<0.001 *	1.5 + 0.7	1.6 + 0.5	0.112
	Constraints	1.7 + 0.3	1.9 + 0.7	0.027 *	2.0 + 0.8	1.7 + 0.5	<0.001 *	1.8 + 0.6	2.0 + 0.8	0.005 *
	Calculation	2.1 + 0.4	3.0 + 1.5	<0.001 *	3.4 + 1.6	2.4 + 1.0	<0.001 *	2.7 + 1.3	3.3 + 1.6	0.001 *
	Collective responsibility	4.7 + 0.2	4.5 + 0.8	0.203	4.5 + 0.8	4.6 + 0.6	0.421	4.6 + 0.7	4.5 + 0.8	0.473

+ JD 1 = USD 1.41; * Statistical significance was set for a *p*-value of less than 0.05.

**Table 4:** Mean barrier and 5C construct score differences across educational level, living arrangement, and marital status.

Measures Domains		Educational Level					Living Arrangement			Marital Status		
		No Education	Less Than 12th Grade	Secondary Education	Graduate or Higher	*p*-Value	In Camp	Outside Camp	*p*-Value	Married	Divorced or Widowed	*p*-Value
**Barrier**	**Access**	39.3 + 16.7	31.8 + 13.6	41.3 + 19.7	35.6 + 14.4	<0.001 *	26.6 + 7.9	42.9 + 17.0	<0.001 *	34.1 + 15.3	42.2 + 17.9	0.056
	**System**	28.6 + 10.9	23.0 + 9.1	29.2 + 13.9	29.3 + 11.0	<0.001 *	19.8 + 5.2	31.4 + 11.9	<0.001 *	25.0 + 10.6	33.4 + 11.7	0.004 *
	**Concerns and Beliefs**	47.6 + 16.9	41.6 + 16.0	53.1 + 22.2	49.2 + 18.4	<0.001 *	35.3 + 9.0	55.2 + 19.5	<0.001 *	44.3 + 17.4	57.9 + 23.4	0.001 *
	**Perceptions**	28.1 + 10.8	23.4 + 9.6	28.6 + 11.9	28.7 + 10.9	<0.001 *	19.4 + 5.4	31.7 + 11.0	<0.001 *	25.0 + 10.4	32.2 + 10.6	0.006 *
	**Knowledge**	22.5 + 10.1	19.3 + 9.2	24.9 + 13.1	25.0 + 11.2	<0.001 *	15.7 + 5.7	27.2 + 11.3	<0.001 *	20.9 + 10.3	28.5 + 11.8	0.009 *
	**Social and Family**	14.3 + 4.5	13.0 + 3.7	14.8 + 4.1	14.7 + 4.0	0.003 *	12.3 + 3.1	15.2 + 4.1	<0.001 *	13.6 + 3.9	15.1 + 4.1	0.118
**Construct**	**Confidence**	6.5 + 0.4	6.7 + 0.4	6.6 + 0.4	6.6 + 0.2	0.009 *	6.7 + 0.5	6.6 + 0.2	0.589	6.7 + 0.4	6.6 + 0.2	0.609
	**Complacency**	1.7 + 0.7	1.6 + 0.7	1.3 + 0.6	1.3 + 0.4	<0.001 *	1.9 + 0.7	1.1 + 0.3	<0.001 *	1.6 + 0.7	1.1 + 0.3	0.003 *
	**Constraints**	2.1 + 0.7	1.9 + 0.8	1.7 + 0.4	1.6 + 0.3	0.003 *	2.0 + 0.8	1.7 + 0.4	<0.001 *	1.8 + 0.7	1.8 + 0.5	0.678
	**Calculation**	2.4 + 1.1	3.1 + 1.5	2.7 + 1.4	2.5 + 1.1	0.013 *	3.6 + 1.6	2.2 + 0.6	<0.001 *	2.9 + 1.5	2.1 + 0.3	0.001 *
	**Collective responsibility**	4.2 + 1.0	4.6 + 0.6	4.4 + 0.8	4.6 + 0.5	0.004 *	4.5 + 0.9	4.6 + 0.3	0.075	4.6 + 0.7	4.6 + 0.4	0.871

* Statistical significance was set for a *p*-value of less than 0.05.

## Data Availability

Data supporting reported results can be found at https://doi.org/10.6084/m9.figshare.25076075.
